# Link Protein N-Terminal Peptide as a Potential Stimulating Factor for Stem Cell-Based Cartilage Regeneration

**DOI:** 10.1155/2018/3217895

**Published:** 2018-01-30

**Authors:** Ruijun He, Baichuan Wang, Min Cui, Zekang Xiong, Hui Lin, Lei Zhao, Zhiliang Li, Zhe Wang, Shaun Peggrem, Zhidao Xia, Zengwu Shao

**Affiliations:** ^1^Department of Orthopaedics, Union Hospital, Tongji Medical College, Huazhong University of Science and Technology, Wuhan 430022, China; ^2^Centre for NanoHealth, College of Medicine, Swansea University, Singleton Park, Swansea SA2 8PP, UK

## Abstract

**Background:**

Link protein N-terminal peptide (LPP) in extracellular matrix (ECM) of cartilage could induce synthesis of proteoglycans and collagen type II in cartilaginous cells. Cartilage stem/progenitor cells (CSPCs), the endogenous stem cells in cartilage, are important in cartilage degeneration and regeneration. We hypothesized that LPP could be a stimulator for stem cell-based cartilage regeneration by affecting biological behaviors of CSPC.

**Methods:**

CSPCs were isolated from rat knee cartilage. We evaluated the promoting effect of LPP on proliferation, migration, and chondrogenic differentiation of CSPCs. The chondrogenic differentiation-related genes and proteins were quantitated. Three-dimensional culture of CSPC was conducted in the presence of TGF-*β*3 or LPP, and the harvested pellets were analyzed to assess the function of LPP on cartilage regeneration.

**Results:**

LPP stimulated the proliferation of CSPC and accelerated the site-directional migration. Higher expression of SOX9, collagen II, and aggrecan were demonstrated in CSPCs treated with LPP. The pellets treated with LPP showed more distinct characteristics of chondroid differentiation than those with TGF-*β*3.

**Conclusion:**

LPP showed application prospect in cartilage regeneration medicine by stimulating proliferation, migration, and chondrogenic differentiation of cartilage stem/progenitor cells.

## 1. Introduction

Articular cartilage is insufficient with intrinsic repairing ability because of its avascular and nerveless structure. Either sizable trauma or accumulated injury can impair articular cartilage, leading to degeneration diseases that cause symptoms and impact living quality [1].

For more efficient repair of the cartilage, the regenerative medicine provides a variety of trials. While the ability in forming autologous cartilage is the gold standard for seed cells in the cartilage tissue engineering, cartilage stem/progenitor cell (CSPC), a subpopulation located in both normal and degenerated human joints [2–4], inspired high hopes for its in situ repairing potency. It participates in the maintenance of homeostasis in the process of cartilage degeneration [5] and meets the requirement of seed cells for cartilage regeneration.

Intra-articular injection of growth factor is thought to be useful in cartilage repair. Bone morphogenetic proteins (BMPs) [6], transforming growth factor-*β*3 (TGF-*β*3) [7], insulin-like growth factor-1 (IGF-1) [8], and so on have been investigated in previous researches. But these growth factors have also shown osteoinductive activity on cartilage tissue [9, 10], which is harmful for long-term effect. As the most common growth factor applied in cartilage tissue engineering, TGF-*β*3 has a short half-life, which results in a dilemma that when it existed in the joint for too long, TGF-*β*3 would enhance inflammation [9], but a short-term application may not be sufficient for cartilage regeneration. The potential oncogenicity is also a disadvantage for the clinical application of TGF-*β*3 [11]. Link protein, a glycoprotein that exists in human intervertebral discs as well as in the articular cartilage [12], plays an important role in strengthening the binding between aggrecan and hyaluronan [13]. Link protein N-terminal peptide (LPP) is the cleaved N-terminal 16 amino peptide (DHLSDNYTLDHDRAIH) of link protein. LPP was thought to be the functional fragment of link protein as the cross-linker [14, 15]. Both native and biochemistry-synthesized form of LPP have an effect on promoting the production of collagen type II and proteoglycans in human intervertebral disc [16] and other cartilaginous cells in vitro [17, 18], which make it an alternative for cartilage regeneration medicine.

In this study, we focused on the effects of LPP on proliferation, migration, and chondrogenic differentiation of rat cartilage stem/progenitor cells to see if LPP can act as a stimulating factor for the CSPC-based cartilage regeneration.

## 2. Materials and Methods

### 2.1. Peptide Synthesis

Peptide LPP (DHLSDNYTLDHDRAIH) representing the consensus sequence of human N-terminal link protein was synthesized following the standard solid phase peptide synthesis method by Zhejiang ONTORES Biotechnologies Co. Ltd (Hangzhou, China). The peptide was characterized by electrospray ionization mass spectroscopy and the purity was higher than 92%. The quality report was shown in Supplementary 1.

### 2.2. Isolation and Culture of CSPCs

All animal studies were conducted in accordance with approved protocols of the Huazhong University of Science and Technology animal experimentation committee. Cells were collected from surface cartilage of female Sprague-Dawley rats (aged 8 weeks, 200 g~250 g) after 8 hours of digestion with 0.25% type II collagenase at 37°C and filtration with a 150 *μ*m strainer. Then, the cells were resuspended in DMEM/F12 (HyClone, USA) and diluted to a density of 4000 mL^−1^. CSPCs were isolated through a fibronectin adhesion assay as described previously [2, 19]. Briefly, 10 cm dishes were coated with 10 *μ*g/mL fibronectin (Sigma, UK) in 0.1 M phosphate-buffered saline (PBS) containing 1 mM MgCl_2_ and 1 mM CaCl_2_ (PBS+) overnight at 4°C. 2 × 10^4 cells were seeded onto the coated plates for 10 min adhesion at 37°C. Then, medium and nonadherent cells were removed. Low glucose DMEM/F12 (HyClone, USA) containing 10% fetal bovine serum (FBS) and 1% penicillin/streptomycin, the complete culture medium, was added into the dishes and renewed every other day for further culture. When it reached 80%~90% confluence, cells were digested by 0.25% trypsin plus 0.02% EDTA (Gibco, USA) for analysis or expanding culture.

### 2.3. Flow Cytometry

Passage 1 of cartilage stem/progenitor cells that resuspended in PBS at a concentration of 1.0 × 10^6 mL^−1^ were aliquoted into 200 *μ*L in test tubes (BD352052, USA) and centrifuged at 1500 rpm, 5 minutes to remove the trypsin and EDTA. The resulting cells were incubated with a solution of antibody CD90, CD73, CD105, CD44, CD34, or HLA-DR at 37°C for 30 min. Besides, another sample was resuspended with PBS and set as calibration. The labeled cells were rinsed and then marked by secondary antibodies at 37°C for 15 minutes. The information of antibodies used in this study was shown. Cells were washed twice and resuspended in 200 *μ*L FACS-Buffer prior to their flow cytometry analyses with FACS Calibur (Becton Dickinson and Company, USA). The information on antibodies is shown in Table 1.

### 2.4. Multilineage Differentiation Potential Assay

CSPCs were seeded onto 24-well plates at a concentration of 2 × 10^4 per well. For osteogenic differentiation, when the cells reached 80%~90% confluence, the culture media was replaced by 0.5 mL osteogenic differentiation induction medium (Cyagen Biosciences Inc., USA) containing 10% FBS, 10 nM dexamethasone, 10 mM *β*-glycerophosphate, and 0.1 mM L-ascorbic acid-2-phosphate. Induction medium was replaced each other day. After four weeks, the cells were rinsed and fixed by 4% formaldehyde. Alizarin red staining was performed following the instruction of the manufacturer.

For adipogenic differentiation, medium was changed into adipogenic differential medium A (purchased from Cyagen Biosciences Inc., USA) for induction when the cultures reached full confluence. Three days later, adipogenic differential media B (Cyagen Biosciences Inc., USA) was used for 24-hour maintaining. Four cycles later, cells were incubated with adipogenic differential media B for 6 more days. Oil Red O (Beyotime, China) staining was used for lipid detecting, which would show lipid droplets in reddish-brown.

### 2.5. Cytotoxicity and Proliferation Assay of LPP

Cell Counting Kit-8 (Dojindo, Japan) assay was performed in 96-well plates with different CSPC seeding density to decide optimal protocols for the following experiments. For cytotoxicity test, CSPCs were seeded on 96-well plate at a density of 1 × 10^4 per well and incubated in 10% FBS culture medium overnight. After adhesion, cells were then treated with various concentrations of LPP in serum-free medium. For proliferation assay, however, 1 × 10^3 cells were seeded into each well and cultured in 10% FBS medium containing LPP. In both experiments, medium was changed every other day. Cell Counting Kit-8 assays were performed at 1st, 3rd, and 5th day after the cell seeding.

### 2.6. Scratch Assay

CSPCs were seeded onto six-well plates and cultured to a confluent monolayer. After making a “scratch” in the middle of cell layer with a 200 *μ*L pipette tip, the cultures were rinsed gently with PBS to discard debris. The cells were cultivated in serum-free medium with or without LPP for 48 hours. Migration was documented by phase contrast microscopy and the photos were analyzed with a computer program.

### 2.7. Chemotaxis Tests

Effect of LPP as chemokine was analyzed via a 24-well microchemotaxis chamber (Corning, USA) with 8 *μ*m-pore polycarbonate filters. LPP was added into the lower compartment of the chemotaxis chamber at a concentration of 10 ng/mL or 100 ng/mL in serum-free medium. In another group, medium was supplemented with 10 ng/mL TGF-*β*3 (PeproTech Inc., USA) to provide comparison. DMEM/F12 in the lower well served as a negative control (basal migration) for each experiment. The upper chambers were loaded with 100 *μ*L cell suspension (2 × 10^4 cells) and incubated at 37°C, 5% CO2 in 95% humidity. After 24 hours, the filters were taken out and washed gently with PBS. The nonmigrated cells on the upper chamber were removed by cotton swabs. Those on the lower side, which were regarded as migrated cells, were fixed with 4% formaldehyde and stained with crystal violet. The number of migrated cells were counted under an inverted microscope.

### 2.8. Real-Time PCR Analysis

Different concentration of LPP was added into the culture medium for 7 days of incubation. Total RNA in different groups was extracted with TRIzol (Invitrogen, USA). The cDNA was obtained with Revert Aid Reverse Transcriptase (Fermenta, EP0442) according to manufacturer's instructions. The expression levels of chondrogenic genes (aggrecan, collagen II, and SOX9) as well as osteogenic markers (Runx2 and collagen X) were quantified. Real-time polymerase chain reactions (RT-PCRs) were performed using the ABI StepOnePlus Real-Time PCR System (Applied Biosystems, Foster City, CA) and KAPA SYBR® FAST qPCR Kit (Kapa Biosystems, USA). Relative RNA quantities were normalized to the amounts of the endogenous control gene, *β*-actin. The results are represented as a ratio to control group using the comparative Ct method of relative quantification. The sequences of primers used in qPCR are presented in Table 2.

### 2.9. Western Blot Analysis

CSPCs were treated with different concentration of LPP for 7 days. Protein from the culture medium as well as cell lysates in each group was collected after preparation with lysis buffer (RIPA Lysis Buffer, Beyotime, China) on ice. Lysate proteins were separated with SDS-PAGE and transferred to PVDF membranes following the routine mentioned before [20]. Primary antibodies against SOX9 (Abcam, USA), collagen type II (Novus Biologicals, USA), aggrecan (Novus Biologicals, USA), or *β*-actin (Tianjin Sungene Biotech Co., China) were applied to blots labeling at 4°C overnight. The blots were then incubated with secondary antibodies (Boster Biological Technology Co. LTD., China) for 1 h. The bands were visualized by the enhanced chemiluminescence (ECL) procedure (Amersham Biosciences, Piscataway, NJ, USA) and integrated density was quantified using Image J software.

### 2.10. Chondrogenic Differentiation Assay

CSPCs were aliquoted into 500 *μ*L DMEM/F12 of 2.5 × 10^5 cells. Suspensions were centrifuged at 1500 rpm, 5 minutes in 15 mL tubes to form spherical pellets which were then immersed by 0.5 mL incomplete culture medium (Cyagen Biosciences Inc., USA) containing ITS (10 mg/mL insulin, 5.5 mg/mL transferrin, and 5 ng/mL selenium), 100 mg/mL gentamicin, 50 mg/mL L-ascorbic acid, 2 mM L-glutamine, 10 mM HEPES, 10^-7 M dexamethasone, and 2% FBS. To evaluate the effect of LPP on chondrogenic differentiation of CSPC, groups were set as control (no LPP neither TGF-*β*3), LPP (50 ng/mL), TGF-*β*3 (10 ng/mL), and LPP (50 ng/mL) + TGF-*β*3 (10 ng/mL). During the 14 days of induction, the medium was changed every other day. Pellets were photographed for general observation and then subjected to histology and immunohistochemistry analysis after formalin fixation and paraffin embedding.

### 2.11. Pellet Analysis

The sections of each pellet were dewaxed with xylene, hydrated in a decreasing graded alcohol series, and then washed three times with dH_2_O. Toluidine blue staining and safranin O staining were conducted for proteoglycan detection and extracellular matrix assessment following conventional methods.

For immunohistochemistry tests, antigen retrieval was conducted and the endogenous peroxidase and heterogenetic antigen were blocked by 3% H_2_O_2_ and 10% goat serum, respectively. Sections were incubated with antibody of collagen II (Abcam, USA) and SOX9 (Abcam, USA) at 4°C overnight. The labeled sections were submitted to Dako REALTM EnVision Detection System following the instruction of the manufacturer. The secondary antibody was set as negative control. Haematoxylin was used for nuclear staining. After coloration, sections were observed with inversion microscope.

### 2.12. Statistical Analysis

All data are reported as mean ± standard deviation (SD) from at least three independent technical replicates. Statistical analysis was performed using SPSS 18.0. The differences between the two groups were analyzed with Student's *t*-tests. Multiple data were analyzed by one-way analysis of variance (ANOVA) followed by the least significant difference (LSD). Statistically significant differences were considered when *p* < 0.05.

## 3. Results

### 3.1. Identification of Cartilage Stem/Progenitor Cells

**Figure 1:**
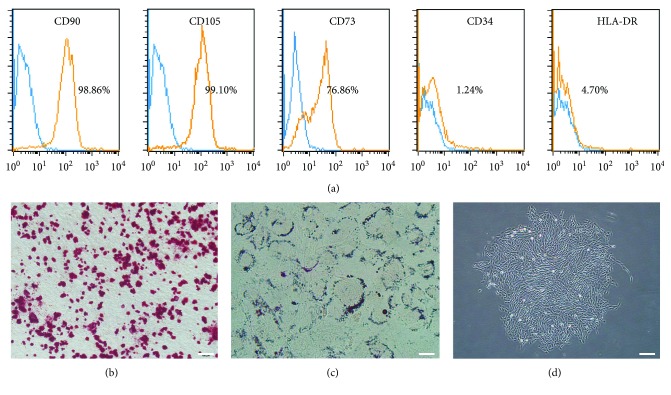
Identification of CSPCs. (a) Flow cytometric analysis for CSPC surface markers. Data were shown as percentage of deviation from the unlabeled groups (blue curve). (b) The alizarin red staining showed calcium nodes after osteogenic differentiation. Bar = 100 *μ*m. (c) The Oil Red O staining showed lipid droplets after adipogenic differentiation. Bar = 20 *μ*m. (d) Morphology of the cartilage stem/progenitor cells under an inverted microscope. Bar = 200 *μ*m.

The cell surface markers of CSPCs were analyzed by FACS. High levels of CD90 and CD105 (both > 90%) were detected. CD73 was also positive (>75%) while almost no expression of CD34 or HLA-DR was observed (Figure 1(a)). The multilineage differentiation potency was tested with osteogenic and adipogenic induction. Safranin O staining displayed the calcium nodes in red (Figure 1(b)) and the Oil Red staining showed lipid droplets that distributed around the edge of cytoplasm (Figure 1(c)). CSPCs grew in a fibroblast cell-like shape and were able to form colonies from one single cell, for the culture density was extremely low (Figure 1(d)). While CSPC was a subpopulation isolated from articular cartilage and represented progenitor status of chondrocytes, we thought that chondrogenic differentiation test was helpless to stem cell identification and the results were not shown. Comparing with the experiment conducted by other researchers [21, 22], we confirmed that the characteristics of these cells conform to the criteria of International Society for Cellular Therapy (ISCT) [23].

### 3.2. The Effect of LPP on Cell Viability of CSPCs

**Figure 2:**
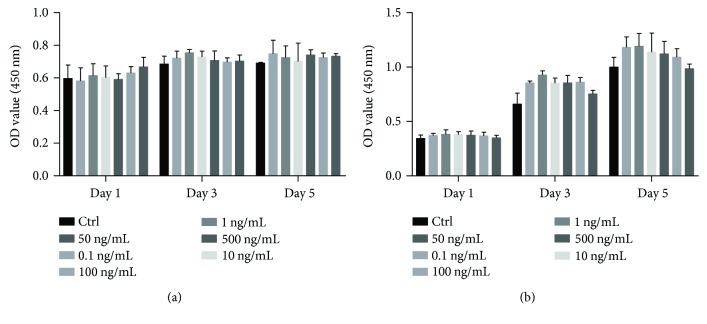
Toxicity tests (a) and proliferation test (b) were performed with CCK-8 kit. OD value at 450 nm was analyzed at the 1st, 3rd, and 5th days, respectively. Results were shown as means ± SD. The data of experimental groups were compared with each associated control group. ^∗^ *p* < 0.05; ^∗∗^ *p* < 0.01.

The growth of CSPCs which were seeded in 96-well plates in various densities was analyzed with CCK-8 assays after the 1, 2, and 3 days of culturing. Briefly, the CSPCs were grown slowly at 100, 500, and 1000 cells per well but proliferated distinctly when seeded 5000 or 10,000 cells per well. Results were shown in Fig supp 1. Therefore, we seeded 10,000 cells per well to see if LPP would impair the cell viability or suppress the proliferation for toxicity test. In order to investigate the promoting effect of LPP on the proliferation of CSPC, 1000 cells were seeded per well for the following tests. During the 5 days of culture, cell viability was not influenced by LPP (Figure 2(a)). Meanwhile, the proliferation of CSPCs was promoted by LPP at a concentration of 0.1 ng/mL and 1 ng/mL in the third day, *p* < 0.05 (Figure 2(b)). The promoting effect with 0.1 ng/mL and 1 ng/mL LPP was sustained on the fifth day (*p* < 0.01), indicating that LPP accelerates the proliferation of CSPCs continuously. In the fifth day, 10 ng/mL LPP also showed a stimulation effect, *p* < 0.05. Although the OD value in the LPP groups at third day and fifth day implied an upgoing trend when compared to the control group, the *t*-tests showed no significant difference between groups of higher concentration LPP and the control.

### 3.3. LPP Promoted the Migration of CSPCs

**Figure 3:**
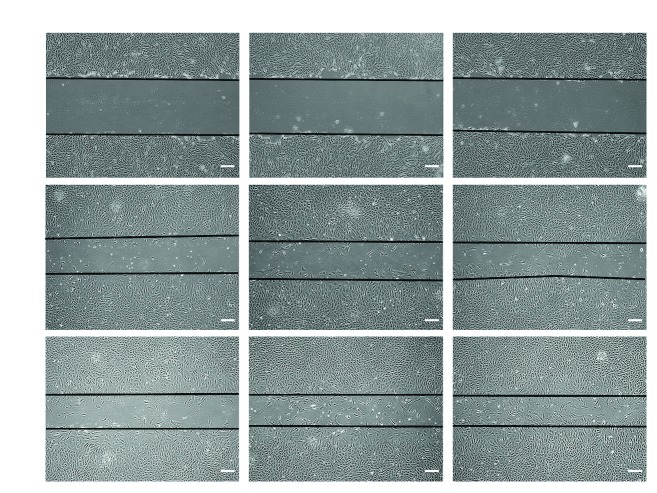
Scratch assays. The migration of CSPCs was documented with an inverted microscope at 0 hour, 24 hours, and 48 hours after the “scratch.” The black lines indicate the baseline from which the cells migrated. Bar = 50 *μ*m.

Confluent CSPCs were scratched and incubated in serum-free culture medium for 48 hours. LPP was added at a concentration of 10 ng/mL and 100 ng/mL. The migration of each group at 0 hour, 24 hours, and 48 hours after scratch is depicted in Figure 3. The black lines indicate the starting line from which the cells crawled during the 48 hours of incubation. In the control group, few cells existed between the black lines. However, when the medium was supplemented with LPP, CSPCs infiltrated the gap and LPP at 100 ng/mL accelerated this process comparing to 10 ng/mL. It is notable that 48 hrs later, the scratch in 100 ng/mL LPP was almost closed with an indistinctive shrinking in the control group.

**Figure 4:**
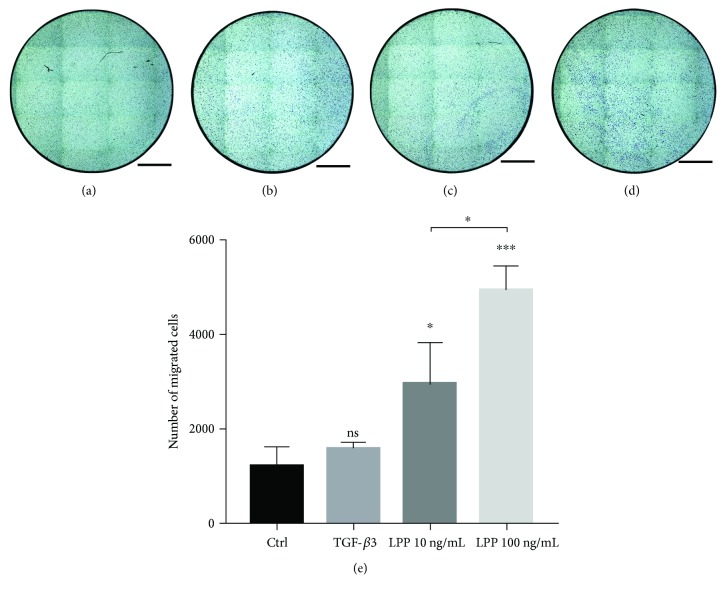
Transwell assay. Image synthesis was performed with EVOS™ FL Auto 2 Imaging System. (a) Control group, (b) TGF-*β*3, (c) LPP 10 ng/mL, and (d) LPP 100 ng/mL. Bar = 1 mm. (e) Number of migrated cells. Cells were counted with a computer program and analyzed with t-tests. ^∗^ *p* < 0.05; ^∗∗∗^ *p* < 0.001.

We then performed transwell test to compare site-directional chemotaxis effect of TGF-*β*3 and LPP. After 48 hours incubation, the chambers were taken out and cells were stained with crystal violet. The lower side of the filtration membrane was photographed and montaged into a panorama (Figure 4). The violet spots represent cells that crossed through the micropore, displaying a tendency of numbers of migrated cells, A < B < C < D. Numbers of migrated CSPC were calculated and analyzed with the method mentioned above, Figure 4(e). In the control group, 1219 ± 404 cells went through the filtration, while 2946 ± 882 cells in LPP (10 ng/mL) and 4931 ± 504 cells in LPP (100 ng/mL) traversed to the lower side. There were 1586 ± 129 cells in TGF-*β*3 group, but the difference was without significant meaning when compared to the control group, *p* > 0.05.

### 3.4. LPP Regulated the Expression of Chondrogenic-Related Genes and Synthesis of Matrix

**Figure 5:**
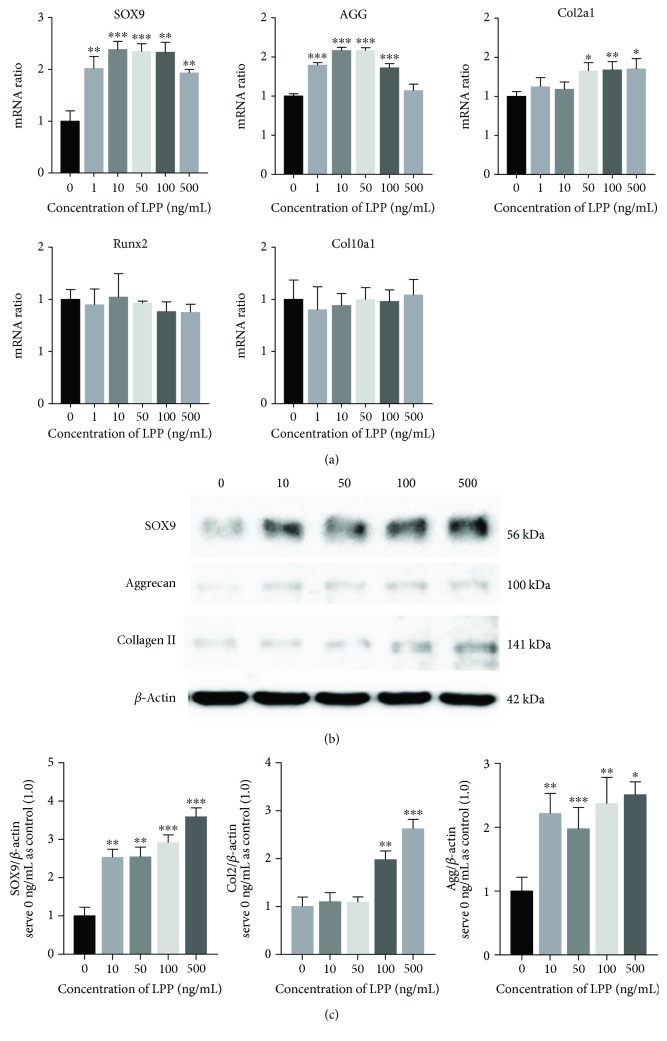
(a) Relative mRNA levels of SOX9, aggrecan, collagen type II, Runx2, and collagen type X in different concentration of LPP. Error bars are means ± SD. (b) Bandings of SOX9 and collagen type II represented the protein content of cells treated with different concentration of LPP. (c) Integrated density of each banding was shown in columns as means ± SD. Data of experimental groups were compared with the control group (0 ng/mL) using Student's *t*-test, respectively. ^∗^ *p* < 0.05; ^∗∗^ *p* < 0.01; ^∗∗∗^ *p* < 0.001.

To figure out if LPP can influence the chondrogenic differentiation and matrix synthesis of CSPCs, cells were treated with different concentration of LPP in monolayer culture. The qPCR tests demonstrated that the expression of SOX9, which is thought to be related with chondrogenesis, was promoted by LPP at doses of 1, 10, 50, 100, and 500 ng/mL compared to untreated cells (0 ng/mL). For aggrecan, the group of 500 ng/mL showed no significant difference while others upregulated the expression, *p* < 0.001. The mRNA ratio of collagen II was influenced by 50, 100, and 500 ng/mL LPP (Figure 5(a)). Thus, 50 ng/mL LPP was used in following experiments: fold change of SOX9 was 2.34 ± 0.15, aggrecan was 1.58 ± 0.04, and col2 was 1.32 ± 0.11. We also found that the mRNA levels of Runx2 and collagen X were not affected by LPP, indicating that LPP had no effect on osteogenic induction (Figure 5(a)). Western blot assays semiquantified the protein levels of SOX9, collagen type II, and aggrecan in cells incubated with LPP (Figure 5(b)). The integrated optical density of each banding was analyzed by setting *β*-actin as an internal reference (Figure 5(c)). SOX9 and aggrecan were apparently elevated by LPP, while collagen II was enhanced only in 100 and 500 ng/mL LPP.

### 3.5. Pellet Matrix Synthesis with LPP or TGF-*β*3

**Figure 6:**
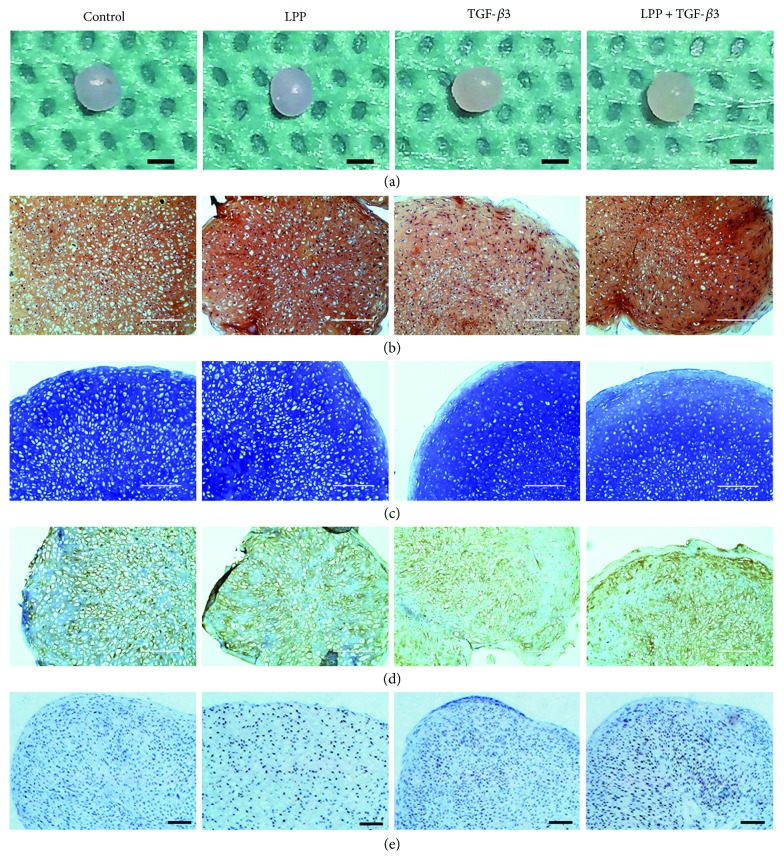
Pellets formed by CSPCs after 2-week chondrogenic differentiation induction. (a) General view of pellets. Bar = 1 mm. (b) Safranin O staining of sections (thickness is less than 2 *μ*m). Bar = 200 *μ*m. (c) Toluidine blue staining of sections. Bar = 200 *μ*m. (d) Immunohistochemistry test of collagen type II. The collagen type II was labeled in yellow. Bar = 200 *μ*m. (e) SOX9 was labeled in brown in IHC assays. Bar = 200 *μ*m.

CSPCs were aliquoted and centrifuged to form pellets. The chondrogenic differentiation basal medium was prepared and supplemented with 50 ng/mL LPP or 10 ng/mL TGF-*β*3 or both. After 14 days, pellets from four groups all showed a smooth surface morphology (Figure 6(a)). The size of LPP + TGF-*β*3 is bigger than others and got a more spherical shape. Safranin O and toluidine blue staining of the paraffin sections of pellets demonstrated glycosaminoglycan synthesis after the induction (Figures 6(b) and 6(c)). When compared to control, the slices in LPP and LPP + TGF-*β*3 groups were stained in more thick jacinth in Figure 6(b), indicating stimulating effects on matrix synthesis. At the edge of pellets, the majority of aggrecan stained by toluidine blue was distributed (Figure 6(c)), and the density of CSPCs was obviously lower in TGF-*β*3 and LPP + TGF-*β*3 groups, indicating more abundant extracellular matrix synthesis (Figure 6(c)). Immunohistochemical methods were also performed to assess the synthesis and location of collagen type II (Figure 6(d)) and SOX9. Collagen II in LPP and LPP + TGF-*β*3 groups is more than in control. In the pellet incubated with LPP, collagen II presented in an approximately radial distribution pattern. IHC of SOX9 in Figure 6(e) showed that LPP enhanced expression of SOX9. In LPP + TGF-*β*3 group, however, this increase was distinctly improved when compared to TGF-*β*3 group, indicating that LPP have the potency of stimulating chondrogenic differentiation of CSPCs, and it can also promote the inducing effect of TGF-*β*3.

## 4. Discussion

CSPCs were isolated from normal and degenerative human cartilage as well as rat knee joints [24]. It was thought to participate in the renewal of chondrocytes and cartilage extracellular matrix [5, 25]. To modulate the regenerative function of CSPC, varieties of factors were tested, for instance, the fibroblast growth factor-2 (FGF-2) [26], TGF-*β*3, IGF [8], and BMPs [27, 28]. LPP is a peptide cleaved from link protein, which exists in cartilage, bridging the collagen type II and hyaluronic acid. It was reported that LPP promoted synthesis of collagen II and proteoglycan in nucleus pulposus cells from intervertebral discs [16, 29] and human chondrocytes [30]. However, the effect of LPP on stem cells, especially the endogenous stem cells in cartilage, remains to be illuminated. In the present study, we evaluated for the first time the effect of LPP on biological behaviors of CSPC, which can favor the stem cell-based cartilage regeneration.

The characterization of passage 1 CSPCs isolated with differential adhesion assay was analyzed. The FACS results indicated that the surface markers of CSPC were similar to bone marrow-derived stem cells (BMSC) [31] and also in agreement with results from other researches [32]. The differentiation induction assays demonstrated the adipogenic and osteogenic potency of CSPC, similar to the results previously reported [19, 33]. Results of cell count kit-8 suggested that LPP promoted cell proliferation at a concentration of 1, 10, and 50 ng/mL. LPP also accelerated the migration of CSPC in scratch assay in 48 hours. The transwell culture showed chemotaxis effect of LPP, indicating that the directional migration of CSPC might be strengthened with higher concentration of LPP. Although TGF-*β*3 showed chemotaxis effect on bone marrow-derived mesenchymal stem cells [34, 35], the outcome in this experiment was the lack of statistic meaning. While cell migration is necessary for stem cell-based intrinsic repair, our study aroused a possibility that LPP could be used in bioactivity materials as a chemotactic factor to help recruitment. The expression levels of chondrogenesis genes were also promoted with LPP and the results of Western blot conformed to the qPCR, which agreed with the findings in previous researches [17, 36, 37]. LPP also showed chondrogenic induction effect on the CSPC in three-dimensional culture. During the chondrogenesis of the skeleton, the condensation of chondroprogenitor cells was vital for chondrogenic differentiation [38, 39]. The chemotaxis effect of LPP might be beneficial to the formation of cell pellet by accelerating the cell condensation, which resulted in higher histology score. Wang et al. verified that LPP upregulated the expression of SOX9 by binding to BMP-RII [29]. We speculated that cartilage stem/progenitor cells, as a transition style of skeleton stem cell during the chondrogenesis [21], had already expressed BMP-RII on the cell membrane. Besides, a hypothesis is that the BMPRII and relevant signal pathway have higher activity than T*β*R-II during the differentiation of CSPC, which demands further investigation.

In the present research, normal rat cartilage was obtained and the cells were isolated for function test in vitro. To figure out whether LPP can help in the recruitment or migration of CSPC in vivo, further studies were required. The difference between LPP and TGF-*β*3 groups in transwell assay and pellet culture requires more detailed explanation, while TGF-*β*3 was also thought to be a stimulating factor in cartilage tissue engineering [40].

LPP may promote the production of tissue-engineered cartilage in vitro by regulating the metabolism of CSPC, making it possible for application in transplant therapy. During the progressing process of arthritis or degeneration, LPP in cartilage is undermined by the inflammation and immunoreaction, which add up to the disbalance of ECM of cartilage [41, 42]. So, the supplement of LPP might be helpful in delaying the articular degeneration. Allowing for limits of exogenous growth factors such as TGF-*β*3 in the in vivo treatment of osteoarthritis, we believe that LPP would be a preferable alternation.

## 5. Conclusion

Collectively, LPP might be a promising stimulating factor for stem cell-based cartilage regeneration for its promoting function on migration, proliferation, and chondrogenic differentiation of cartilage stem/progenitor cell. With benefits of LPP demonstrated in this research, the CSPC-based cellular therapy gives hope to cartilage regeneration medicine, bringing a new research focus which still requires further investigation.

## Figures and Tables

**Table 1:** Anti-rat antibodies used in flow cytometry analysis.

Antibodies	Code number
*Primary antibody*	
CD90	Abcam, ab3105
CD73	BD, 551123
CD105	Abcam, ab11414
CD34	Abcam, ab81289
HLA-DR	Abcam, ab92511
*Secondary antibody*	
Alexa Fluor® 488	Abcam, ab150113
Alexa Fluor 647	Abcam, ab150075

**Table 2:** The primer sequences used for the amplification of CSPC cDNA.

Gene	Primer sequence	Product length
*SOX9*	Forward: 5′AAGAAAGACCACCCCGATTACA3′	122 bp
	Reverse: 5′ GCCTTGAAGATGGCGTTAGGA3′	

*Aggrecan*	Forward: 5′ATCCAGAACCTTCGCTCCAA3′	157 bp
	Reverse: 5′ GGGCTCGGTCAAAGTCCAGT3′	

*Col2a1*	Forward: 5′ ATTGCCTACCTGGACGAAGC3′	160 bp
	Reverse: 5′ TGATGGTCTTGCCCCACTTAC3′	

*Runx2*	Forward: 5′ TTCCTGTGCTCCGTGCTG3′	236 bp
	Reverse: 5′AAAGTGAAACTCTTGCCTCGTC3′	

*Col10a1*	Forward: 5′ TTTGGATAGGGCAGTGCTTCA3′	239 bp
	Reverse: 5′ CACCCCATCATCAGAGTTCATT3′	

*β-Actin*	Forward: 5′ CGTTGACATCCGTAAAGACCTC3′	110 bp
	Reverse: 5′ TAGGAGCCAGGGCAGTAATCT3′	

## Supplementary Materials

Supplementary 1 The quality report of the synthetic Link protein N-terminal peptide.

Supplementary 2 Proliferation velocity of CSPC in different density. Figure Supplement 1: the growth characteristic of CSPCs. Different numbers of CPSCs were seeded into 96-well plates and cultured in complete culture medium. OD value was detected during the 3-day culturing with CCK-8 assay.

## References

[1] N. C. G. Centre (2014). Osteoarthritis: Care and Management in Adults. *National Institute for Health and Clinical Excellence: Clinical Guidances, No.177*.

[2] Alsalameh S., Amin R., Gemba T., Lotz M. (2004). Identification of mesenchymal progenitor cells in normal and osteoarthritic human articular cartilage. *Arthritis & Rheumatism*.

[3] Dowthwaite G. P. (2004). The surface of articular cartilage contains a progenitor cell population. *Journal of Cell Science*.

[4] Fickert S., Fiedler J., Brenner R. E. (2004). Identification of subpopulations with characteristics of mesenchymal progenitor cells from human osteoarthritic cartilage using triple staining for cell surface markers. *Arthritis Research & Therapy*.

[5] Koelling S., Kruegel J., Irmer M. (2009). Migratory chondrogenic progenitor cells from repair tissue during the later stages of human osteoarthritis. *Cell Stem Cell*.

[6] Noel D., Gazit D., Bouquet C. (2004). Short-term BMP-2 expression is sufficient for in vivo osteochondral differentiation of mesenchymal stem cells. *Stem Cells*.

[7] Bian L., Zhai D. Y., Tous E., Rai R., Mauck R. L., Burdick J. A. (2011). Enhanced MSC chondrogenesis following delivery of TGF-β3 from alginate microspheres within hyaluronic acid hydrogels *in vitro* and *in vivo*. *Biomaterials*.

[8] O'Rear L. (2005). Signaling cross-talk between IGF-binding protein-3 and transforming growth factor-β in mesenchymal chondroprogenitor cell growth. *Journal of Molecular Endocrinology*.

[9] van Beuningen H. M., Glansbeek H. L., van der Kraan P. M., van den Berg W. B. (2000). Osteoarthritis-like changes in the murine knee joint resulting from intra-articular transforming growth factor-β injections. *Osteoarthritis and Cartilage*.

[10] Cheng H., Jiang W., Phillips F. M. (2003). Osteogenic activity of the fourteen types of human bone morphogenetic proteins (BMPs). *The Journal of Bone and Joint Surgery-American Volume*.

[11] Principe D. R., Doll J. A., Bauer J. (2014). TGF-β: duality of function between tumor prevention and carcinogenesis. *Journal of the National Cancer Institute*.

[12] Nguyen Q., Liu J., Roughley P. J., Mort J. S. (1991). Link protein as a monitor in situ of endogenous proteolysis in adult human articular cartilage. *Biochemical Journal*.

[13] Hardingham T. E. (1979). The role of link-protein in the structure of cartilage proteoglycan aggregates. *Biochemical Journal*.

[14] Faltz L. L., Caputo C. B., Kimura J. H., Schrode J., Hascall V. C. (1979). Structure of the complex between hyaluronic acid, the hyaluronic acid-binding region, and the link protein of proteoglycan aggregates from the swarm rat chondrosarcoma. *Journal of Biological Chemistry*.

[15] Kimura J. H., Hardingham T. E., Hascall V. C. (1980). Assembly of newly synthesized proteoglycan and link protein into aggregates in cultures of chondrosarcoma chondrocytes. *Journal of Biological Chemistry*.

[16] Petit A., Yao G., Rowas S. A. (2011). Effect of synthetic link N peptide on the expression of type I and type II collagens in human intervertebral disc cells. *Tissue Engineering Part A*.

[17] McKenna L. A., Liu H., Sansom P. A., Dean M. F. (1998). An N-terminal peptide from link protein stimulates proteoglycan biosynthesis in human articular cartilage in vitro. *Arthritis & Rheumatism*.

[18] Liu H., McKenna L. A., Dean M. F. (1999). The macromolecular characteristics of cartilage proteoglycans do not change when synthesis is up-regulated by link protein peptide. *Biochimica et Biophysica Acta (BBA) - General Subjects*.

[19] Williams R., Khan I. M., Richardson K. (2010). Identification and clonal characterisation of a progenitor cell sub-population in normal human articular cartilage. *PLoS One*.

[20] Lin H., Zhao L., Ma X. (2017). Drp1 mediates compression-induced programmed necrosis of rat nucleus pulposus cells by promoting mitochondrial translocation of p53 and nuclear translocation of AIF. *Biochemical and Biophysical Research Communications*.

[21] Jiang Y., Tuan R. S. (2015). Origin and function of cartilage stem/progenitor cells in osteoarthritis. *Nature Reviews Rheumatology*.

[22] Xue K., Zhang X., Qi L., Zhou J., Liu K. (2016). Isolation, identification, and comparison of cartilage stem progenitor/cells from auricular cartilage and perichondrium. *American Journal of Translational Research*.

[23] Dominici M., Le Blanc K., Mueller I. (2006). Minimal criteria for defining multipotent mesenchymal stromal cells. The International Society for Cellular Therapy position statement. *Cytotherapy*.

[24] Kim M., Kim J., Park S. R. (2016). Comparison of fetal cartilage-derived progenitor cells isolated at different developmental stages in a rat model. *Development, Growth & Differentiation*.

[25] Seol D., McCabe D. J., Choe H. (2012). Chondrogenic progenitor cells respond to cartilage injury. *Arthritis & Rheumatism*.

[26] Bianchessi M., Chen Y., Durgam S., Pondenis H., Stewart M. (2016). Effect of fibroblast growth factor 2 on equine synovial fluid chondroprogenitor expansion and Chondrogenesis. *Stem Cells International*.

[27] Lu C.-H., Yeh T.-S., Yeh C.-L. (2014). Regenerating cartilages by engineered ASCs: prolonged TGF-β3/BMP-6 expression improved articular cartilage formation and restored zonal structure. *Molecular Therapy*.

[28] Neumann A. J., Gardner O. F. W., Williams R., Alini M., Archer C. W., Stoddart M. J. (2015). Human articular cartilage progenitor cells are responsive to mechanical stimulation and adenoviral-mediated overexpression of bone-morphogenetic protein 2. *PLoS One*.

[29] Wang Z., Weitzmann M. N., Sangadala S., Hutton W. C., Yoon S. T. (2013). Link protein N-terminal peptide binds to bone morphogenetic protein (BMP) type II receptor and drives matrix protein expression in rabbit intervertebral disc cells. *Journal of Biological Chemistry*.

[30] Liu H., McKenna L. A., Dean M. F. (2000). An N-terminal peptide from link protein can stimulate biosynthesis of collagen by human articular cartilage. *Archives of Biochemistry and Biophysics*.

[31] McCarthy H. E., Bara J. J., Brakspear K., Singhrao S. K., Archer C. W. (2012). The comparison of equine articular cartilage progenitor cells and bone marrow-derived stromal cells as potential cell sources for cartilage repair in the horse. *The Veterinary Journal*.

[32] Bernstein P., Sperling I., Corbeil D., Hempel U., Fickert S. (2013). Progenitor cells from cartilage—no osteoarthritis-grade-specific differences in stem cell marker expression. *Biotechnology Progress*.

[33] Joos H., Wildner A., Hogrefe C., Reichel H., Brenner R. E. (2013). Interleukin-1β and tumor necrosis factor α inhibit migration activity of chondrogenic progenitor cells from non-fibrillated osteoarthritic cartilage. *Arthritis Research & Therapy*.

[34] Zhang S. J., Song X. Y., He M., Yu S. B. (2016). Effect of TGF-β1/SDF-1/CXCR4 signal on BM-MSCs homing in rat heart of ischemia/perfusion injury. *European Review for Medical and Pharmacological Sciences*.

[35] Dubon M. J., Yu J., Choi S., Park K.-S. (2017). Transforming growth factor β induces bone marrow mesenchymal stem cell migration via noncanonical signals and N-cadherin. *Journal of Cellular Physiology*.

[36] Mwale F., Demers C. N., Petit A. (2003). A synthetic peptide of link protein stimulates the biosynthesis of collagens II, IX and proteoglycan by cells of the intervertebral disc. *Journal of Cellular Biochemistry*.

[37] Wang B., Sun C., Shao Z. (2014). Designer self-assembling peptide nanofiber scaffolds containing link protein N-terminal peptide induce chondrogenesis of rabbit bone marrow stem cells. *BioMed Research International*.

[38] Goldring M. B., Tsuchimochi K., Ijiri K. (2006). The control of chondrogenesis. *Journal of Cellular Biochemistry*.

[39] Bhattacharjee M., Coburn J., Centola M. (2015). Tissue engineering strategies to study cartilage development, degeneration and regeneration. *Advanced Drug Delivery Reviews*.

[40] Jayasuriya C. T., Chen Y., Liu W., Chen Q. (2016). The influence of tissue microenvironment on stem cell–based cartilage repair. *Annals of the New York Academy of Sciences*.

[41] Guerassimov A., Zhang Y., Cartman A. (1999). Immune responses to cartilage link protein and the G1 domain of proteoglycan aggrecan in patients with osteoarthritis. *Arthritis & Rheumatism*.

[42] Guerassimov A., Zhang Y., Banerjee S. (1998). Autoimmunity to cartilage link protein in patients with rheumatoid arthritis and ankylosing spondylitis. *The Journal of Rheumatology*.

