# Supplementary material for: Link Protein N-Terminal Peptide as a Potential Stimulating Factor for Stem Cell-Based Cartilage Regeneration
Source: Stem Cells Int. 2018 Jan 30;2018:3217895. doi: 10.1155/2018/3217895 (PMC5831317; doi:10.1155/2018/3217895)
Supplement: Supplementary 1 — The quality report of the synthetic Link protein N-terminal peptide. [file 3217895.f1.pdf]

Quality Inspection Report

**Product Name:**	LPP
**Lot Num:**	P1701342
**Sequence:**	[CH3CONH]- DHLSDNYTLDHDRAIH-[CONH2]
**Molecular Weight:**	1963.07
**Mass Spectral Analysis:**	see attached MS spectrogram
**HPLC Analysis:**	Peptide purity:>92%
**Solubility:**	1 mg/ml in DMSO
**Appearance:**	White lyophilized powder
**Counter Ion:**	Trifluoroacetate
**Date of Mfg:**	02/27/17

Quality Assurance By: Quality Control Department

Date: 02/27/17

Zhejiang Ontores Biotechnologies Co.,Ltd

Address: NO.16 Road of Long Tan Hangzhou,Zhejiang , China, 311121
Email:support@ontores.com Fax: 0571-88863303 Tel: 0571-88607602 http://www.ontoresinc.com

SAMPLE INFORMATION

Sample Name:	P1701342	Acquired By:	System
Sample Type:	Unknown	Sample Set Name:	20170228
Vial:	25	Acq. Method Set:	15% _35% in 20 mins
Injection #:	1	Processing Method:	Default
Injection Volume:	20.00 ul	Channel Name:	2487Channel 1
Run Time:	20.0 Minutes	Proc. Chnl. Descr.:	220 nm
Date Acquired:	2/28/2017 9:19:09 AM CST		
Date Processed:	2/28/2017 9:43:47 AM CST		

Auto-Scaled Chromatogram

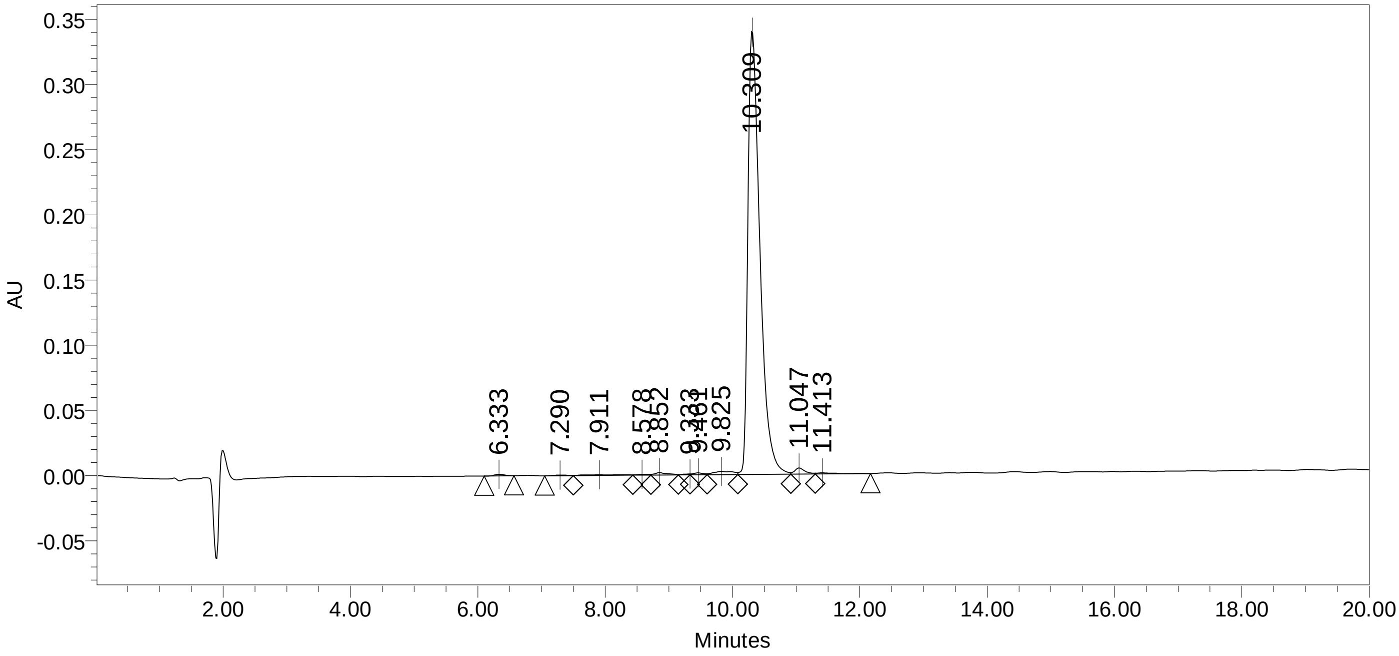

Peak Results

	RT	Int Type	Area	Width (sec)	Height	% Area
1	6.333	bB	13218	28.000	1172	0.28
2	7.290	BV	7377	27.000	422	0.16
3	7.911	VV	21426	56.000	494	0.46
4	8.578	VV	8855	17.000	638	0.19
5	8.852	VV	25728	26.000	1829	0.55
6	9.333	VV	5044	11.000	604	0.11
7	9.461	VV	16223	16.000	1456	0.35
8	9.825	VV	53064	29.000	2453	1.13
9	10.309	VV	4467311	50.000	342193	95.33

Peak Results

	RT	Int Type	Area	Width (sec)	Height	% Area
10	11.047	VV	51834	23.000	4705	1.11
11	11.413	Vb	16249	52.000	816	0.35

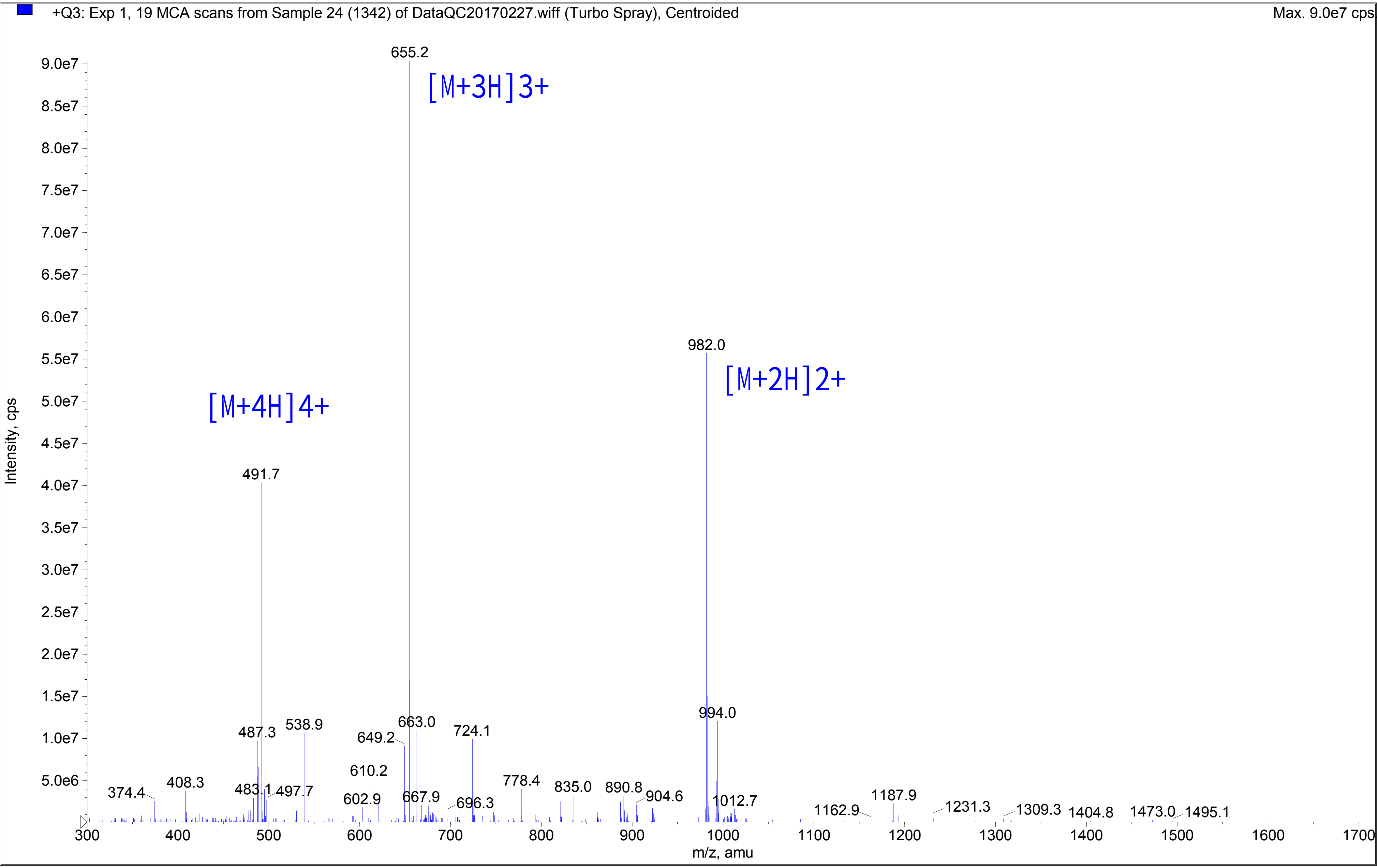

+Q3: Exp 1, 19 MCA scans from Sample 24 (1342) of DataQC20170227.wiff (Turbo Spray), Centroided
Max. 9.0e7 cps
Intensity, cps
m/z, amu
655.2
[M+3H]3+
982.0
[M+2H]2+
491.7
[M+4H]4+
994.0
487.3
538.9
649.2
663.0
724.1
610.2
778.4
835.0
890.8
904.6
374.4
408.3
483.1
497.7
602.9
667.9
696.3
1012.7
1162.9
1187.9
1231.3
1309.3
1404.8
1473.0
1495.1
